# Modified “temporal” sutureless vitrectomy

**DOI:** 10.4103/0301-4738.58489

**Published:** 2010

**Authors:** Cyrus M Shroff, A K Singh, Charu Gupta, Daraius N Shroff

**Affiliations:** Shroff Eye Centre, A -9 Kailash Colony, New Delhi -110 048, India

Dear Editor,

We read with interest the review article “Sutureless Vitrectomy” by Warrier *et al.*[1] We congratulate the authors on their comprehensive review. Among the benefits of sutureless vitrectomy they have listed a decreased intraoperative time, patient discomfort and a much faster healing rate. We would like to add another practical and novel intraoperative benefit of sutureless vitrectomy.

We recently performed a 23-gauge vitrectomy on a 22-year-old phakic lady with a recurrent retinal detachment (RD) who had undergone a previous scleral buckle surgery for a rhegmatogenous RD elsewhere one month prior to presentation. She presented to us with a sub-total RD in the left eye with two very anteriorly placed open retinal breaks in the temporal quadrant, which were anterior to the belt buckle, and were unsupported due to lack of a scleral buckle in that quadrant. She underwent a 23-gauge “modified” trans-conjunctival vitrectomy in which the surgeon (CMS) sat temporally. The inferonasal sclerotomy was used to place the infusion cannula, and the superotemporal and inferotemporal sclerotomies were used for the 23-gauge vitrectomy cutter and light pipe.

This maneuver enabled us to do a more complete vitrectomy with depression around the anteriorly located breaks without having to cross the clear crystalline lens with our vitrectomy cutter and light pipe, hence avoiding the chances of a lens touch. The direction of the port of the vitrectomy cutter could also be correctly aligned with the break through this maneuver.

Sutureless vitrectomy thus gives us the freedom of changing the position of the infusion cannula, even intraoperatively if required and hence, allows us to perform a more complete vitrectomy with greater ease and lesser complications.
